# Otolithic Receptor Mechanisms for Vestibular-Evoked Myogenic Potentials: A Review

**DOI:** 10.3389/fneur.2018.00366

**Published:** 2018-05-25

**Authors:** Ian S. Curthoys, J. Wally Grant, Ann M. Burgess, Chris J. Pastras, Daniel J. Brown, Leonardo Manzari

**Affiliations:** ^1^Vestibular Research Laboratory, School of Psychology, The University of Sydney, Sydney, NSW, Australia; ^2^Department of Biomedical Engineering and Mechanics, VA Tech, Blacksburg, VA, United States; ^3^The Menière’s Laboratory, Sydney Medical School, The University of Sydney, Sydney, NSW, Australia; ^4^MSA ENT Academy Center, Cassino, Italy

**Keywords:** vestibular, utricular, saccular, vestibular-evoked myogenic potential, cervical vestibular-evoked myogenic potential, ocular vestibular-evoked myogenic potential, sound, vibration

## Abstract

Air-conducted sound and bone-conduced vibration activate otolithic receptors and afferent neurons in both the utricular and saccular maculae, and trigger small electromyographic (EMG) responses [called vestibular-evoked myogenic potentials (VEMPs)] in various muscle groups throughout the body. The use of these VEMPs for clinical assessment of human otolithic function is built on the following logical steps: (1) that high-frequency sound and vibration at clinically effective stimulus levels activate otolithic receptors and afferents, rather than semicircular canal afferents, (2) that there is differential anatomical projection of otolith afferents to eye muscles and neck muscles, and (3) that isolated stimulation of the utricular macula induces short latency responses in eye muscles, and that isolated stimulation of the saccular macula induces short latency responses in neck motoneurons. Evidence supports these logical steps, and so VEMPs are increasingly being used for clinical assessment of otolith function, even differential evaluation of utricular and saccular function. The proposal, originally put forward by Curthoys in 2010, is now accepted: that the ocular vestibular-evoked myogenic potential reflects predominantly contralateral utricular function and the cervical vestibular-evoked myogenic potential reflects predominantly ipsilateral saccular function. So VEMPs can provide differential tests of utricular and saccular function, not because of stimulus selectivity for either of the two maculae, but by measuring responses which are predominantly determined by the differential neural projection of utricular as opposed to saccular neural information to various muscle groups. The major question which this review addresses is how the otolithic sensory system, with such a high density otoconial layer, can be activated by individual cycles of sound and vibration and show such tight locking of the timing of action potentials of single primary otolithic afferents to a particular phase angle of the stimulus cycle even at frequencies far above 1,000 Hz. The new explanation is that it is due to the otoliths acting as seismometers at high frequencies and accelerometers at low frequencies. VEMPs are an otolith-dominated response, but in a particular clinical condition, semicircular canal dehiscence, semicircular canal receptors are also activated by sound and vibration, and act to enhance the otolith-dominated VEMP responses.

## Preface

In the last 5 years, there has been a very rapid growth of knowledge concerning vestibular-evoked myogenic potentials (VEMPs) and their physiological basis (1, 2). This includes new understanding of how sound and vibration activate otolithic receptors. The present review seeks to provide a concise comprehensive overview, as accurate as we can make it at May 2018, of the basic physiological mechanisms underlying VEMPs.

## Introduction

**Figure 1:**
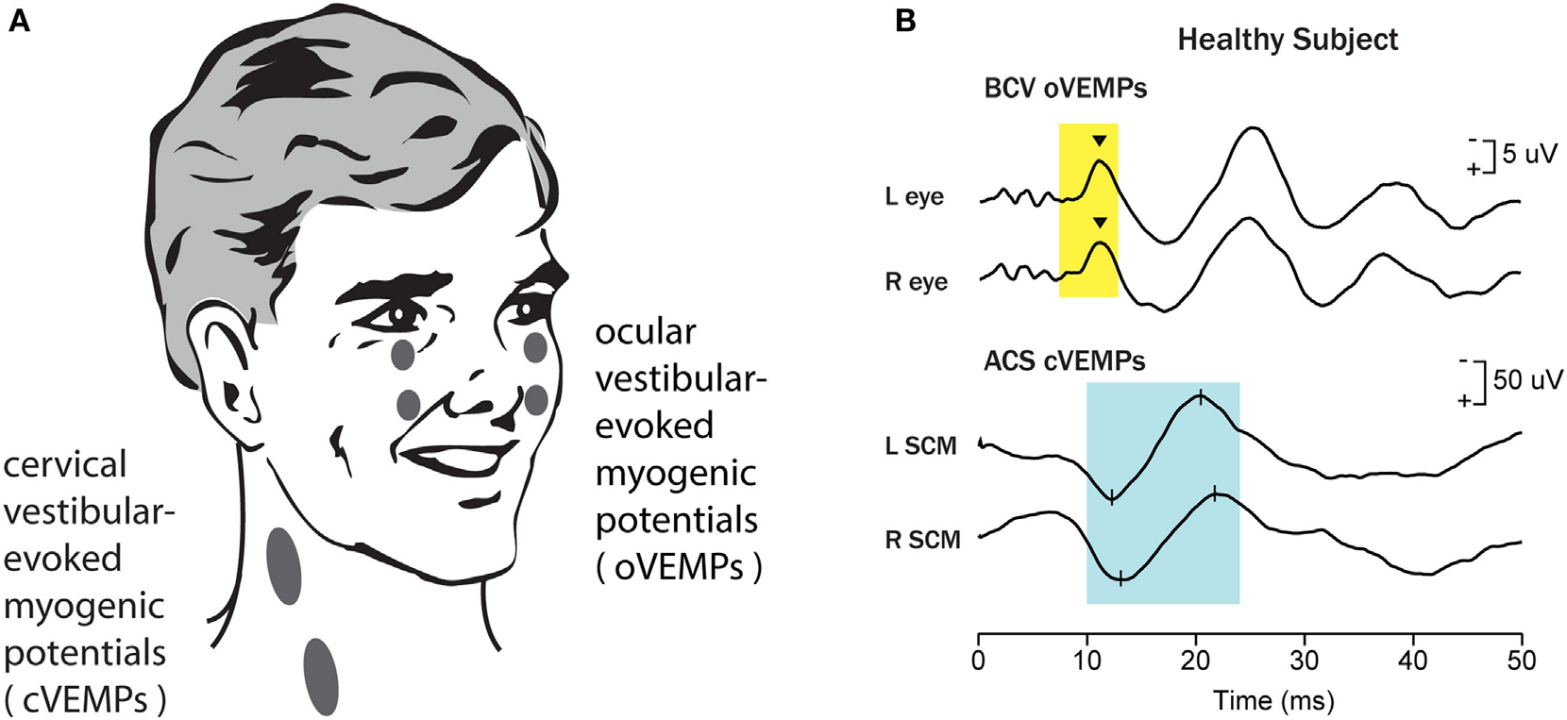
Vestibular-evoked myogenic potentials (VEMPs). There are a host of VEMPs since vestibular input projects indirectly to many muscle groups. The two VEMPs which have received the most attention are cervical vestibular-evoked myogenic potentials (cVEMPs) and ocular vestibular-evoked myogenic potentials (oVEMPs). cVEMPs are recorded by surface electromyographic (EMG) electrodes over the tensed sternocleidomastoid muscles (SCMs) (11). The cVEMP consists of a short latency (13 ms from onset to peak) positive (i.e., inhibitory) EMG potential in response to high-intensity air-conducted sound (ACS) or bone-conducted vibration (BCV) (15). oVEMPs consist of a small (5–10 µV) negative (i.e., excitatory) potential recorded by surface electrodes on the skin beneath the eyes from the inferior oblique in response to BCV or ACS (12, 13). To record the oVEMP, the subject must be looking up. **(A)** Electrode placement for oVEMPs and cVEMPs; the ground electrode (not shown) is typically on the chin or sternum. **(B)** [Reprinted from Iwasaki et al. (16) © 2009, with permission from Elsevier.] Typical oVEMP and cVEMP traces for a healthy subject: the magnitude of the n10 response is approximately equal beneath both eyes for the oVEMP, and similarly the magnitude of the p13–n23 response is approximately equal in both SCMs for the cVEMP.

Before the 1990s, the usual way to probe the function of the otoliths was to measure responses, such as eye movements or perception, to maintained or low-frequency linear acceleration stimuli provided by sleds or centrifuges or tilting chairs (3–8). Such tests were clinically impractical because of the small, variable, unreliable responses, as well as safety issues in delivering the stimuli. Since then there has been a major change: now surface electrodes on the skin are being used to record myogenic potentials in response to sound and vibration to probe otolith function, simply, quickly, reliably, and safely. These are called vestibular-evoked myogenic potentials (VEMPs). It is now clear that because of extensive indirect projections of vestibular neurons there are a host of VEMPs throughout the body (9, 10), with the two most frequently studied being the cervical vestibular-evoked myogenic potential [cVEMP—recorded from above the tensed sternocleidomastoid muscle (SCM)] and the ocular vestibular-evoked myogenic potential (oVEMP—recorded from above the inferior oblique as the patient looks up) (11–14) (see Figure 1).

The primary question is: are VEMP responses to sound (ACS) or bone-conducted vibration (BCV) really due to vestibular activation, since obviously sound and vibration stimulate cochlear receptors? That question was answered by showing the presence of VEMPs in patients without hearing but with vestibular function, and the absence of VEMPs in patients with hearing but without vestibular function after systemic gentamicin (11, 13, 17). These data show conclusively that VEMP tests are vestibular and not cochlear, and that evidence is supported by physiological research showing primary otolithic neurons are activated by sound and vibration. However, the next major question is how sound and vibration activate otolithic receptors and afferents, and that is the main focus of this review—the physiological basis for using these myogenic potentials to index otolith function, and the rationale for using these tests to test utricular or saccular function differentially.

**Figure 2:**
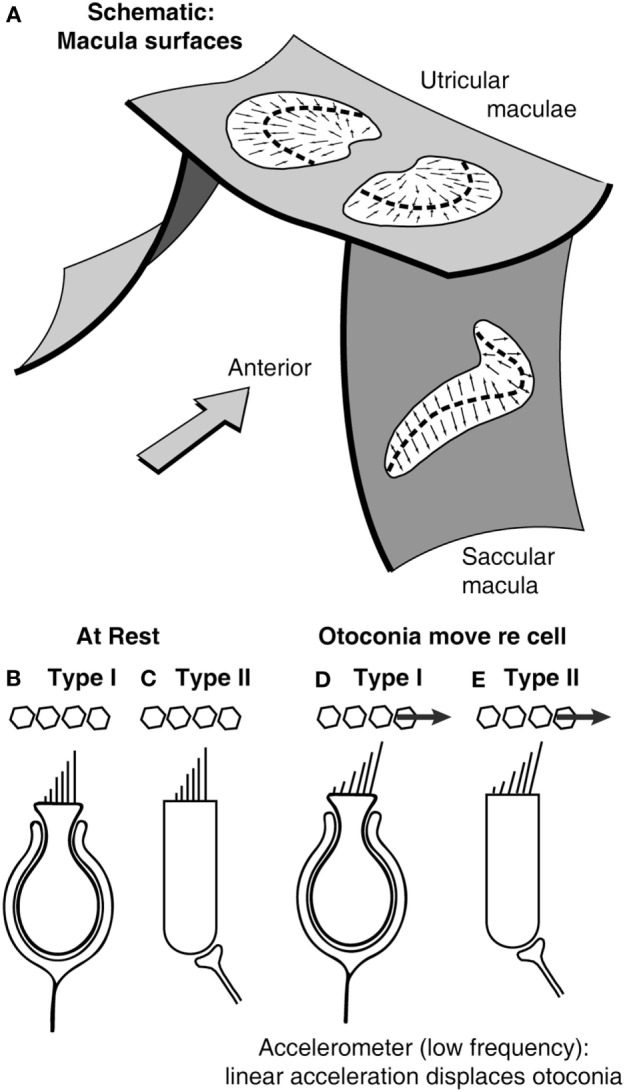
**(A)** Schematic representation of the plates of otolithic receptors (the utricular and saccular maculae). The arrows show the preferred polarization of hair cell receptors across the maculae. The dashed lines are lines of polarity reversal (lpr). The striola refers to a band of receptors on either side of the lpr (27). Schematics of type I **(B,D)** and type II receptors **(C,E)** show how linear acceleration acts on otoliths and so deflects the hair bundles of individual receptors.

The traditional view of the otoliths has been that they are flat sheets of tissue (called maculae—Figure 2) in which there are embedded thousands of receptor hair cells with their hair bundles projecting into the gelatinous otoconial layer (OL) covered by crystals of dense otoconia [specific gravity of 2.73—similar to granite (18)]. In the human, there are around 33,000 receptors in each utricular macula and 18,000 in each saccular macula, with about 5,000 utricular afferents and 4,000 saccular afferents (19, 20). The stimulus for causing vestibular hair cell transduction is deflection of the hair bundle with respect to the cell body of the receptor in the neuroepithelial layer (NEL) of the macula or crista. The traditional view is that the otoliths are stimulated by linear accelerations (such as head tilt) because the linear acceleration drags the otoconia and so deflects the hair bundles of the otolithic receptors (21) (Figures 2B–E). Because of the high density of the otoconia, the otoliths have been regarded as a sensory system responsive to static tilts and fairly low frequencies of linear acceleration—up to a few hundred Hertz [e.g., Ref. (21–26)].

Physiological evidence shows the otoliths do respond to maintained tilts and low-frequency linear accelerations, and here, we call this the accelerometer mode of otolith operation. But there is now abundant evidence that some otolithic receptors and afferents can be activated by air-conducted sound (ACS) and BCV up to frequencies of thousands of Hertz. This is shown by neural recordings of otolithic afferents with irregular resting discharge in squirrel monkey, cat, rat, and guinea pig (28–41). This neural evidence of otolithic activation by high frequencies is the foundation on which VEMPs to ACS and BCV are used to test otolith function.

The maculae are (moderately) curved structures (Figure 2) (42). The receptor cells, embedded in the neurepithelium of the maculae, fall into two types: amphora-shaped type I receptors or cylindrical type II receptors, and these two types are intermingled across the maculae (43, 44). Otolithic receptors are activated by hair bundle deflection toward the longest cilium (the kinocilium), and so each receptor has a preferred direction which is termed its morphological polarization. The receptors have opposite morphological polarization on either side of a dividing line now called “the line of polarity reversal” (Figure 2).

The receptors in a band (called the striola) straddling the line of polarity reversal are especially important—they have short stiff cilia with tenuous attachment to the otoconial membrane (27, 45), and there is a greater concentration of type I receptors in the striola (44, 46). The type I receptor cell bodies are enveloped by the calyx ending of afferents with irregular resting discharge (31, 47, 48). It appears that the attachment of the hair bundles of striolar receptors to the overlying otoconial membrane is tenuous (27, 49, 50). Extracellular recordings from primary otolithic afferents with irregular resting discharge have shown that these afferents are sensitive to sound and vibration, and histological tracing has shown these afferents contact type I receptors at the striola (31, 33).

Songer and Eatock (51) used intracellular recording from isolated type I otolithic receptors and showed that mammalian type I receptors could respond to displacements at frequencies of hundreds of Hertz (and probably higher). The size of these displacements is small, but only small displacements are needed since individual vestibular receptors are almost as sensitive as individual cochlear receptors. Using intracellular recordings from individual receptors stimulated by hair bundle deflection, Geleoc et al. (52) have shown that isolated vestibular receptors have similar thresholds for hair bundle displacement as cochlear receptors—deflections of the receptor hair bundles of around 10 nm generate intracellular potentials in both cochlear and vestibular receptors.

Throughout both utricular and saccular maculae, there are receptors (probably cylindrical type II receptors) with long cilia projecting into the otolithic membrane (45). Afferent neurons with regular resting discharge make extensive contacts with extrastriolar type I and II receptors (47), but guinea pig otolithic afferents with regular resting discharge do not respond to ACS and BCV at reasonable levels [2 *g* peak to peak max or 130 dB sound pressure level (SPL) (31)].

**Figure 3:**
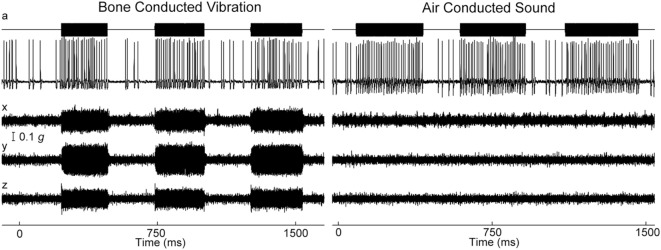
Time series of firing of an irregular otolith neuron during stimulation by bone-conducted vibration (BCV) and air-conducted sound (ACS) at 500 Hz—both stimuli cause stimulus-locked activation. The top trace (a) shows the command voltage indicating when the stimulus is on. The second trace shows the extracellular recording. The three bottom traces (*x, y, z*) show the triaxial accelerometer recording of the stimulus. The *left panel* is an example of BCV stimulation and the *right* of ACS stimulation of the same neuron. Note the scale of stimulus intensity in *g* at the left margin between traces *x* and *y*. Reprinted by permission from Springer Nature, Curthoys and Vulovic (29), © 2011.

Extracellular recordings of single primary otolithic afferents with irregular resting discharge show that they have a stimulus-locked increase in firing rate to ACS or BCV stimulation up to frequencies of thousands of Hertz (31) (Figure 3). The threshold as a function of frequency is very different for ACS vs BCV. For ACS, the lowest thresholds are at around 90 dB SPL at 1,000 Hz, with cells still responding with relatively low thresholds to 2,000 and 3,000 Hz ACS stimuli. For BCV, the lowest threshold is around 0.02 *g* at frequencies from 100 to 500 Hz. For BCV frequencies above 750 Hz, there is a very steep increase in threshold beyond 750 Hz, so that few neurons are activated by BCV at 2,000 Hz (even at 2 *g* peak to peak). At lower frequencies such as 500 and 750 Hz, BCV is a much more effective and reliable stimulus than ACS—the threshold for single neurons to BCV is around 0.02 *g* peak to peak, which is around the level for auditory brainstem response (ABR) threshold, whereas vestibular neural thresholds for ACS are at levels about 70 dB above ABR threshold (29, 31).

## Phase Locking

**Figure 4:**
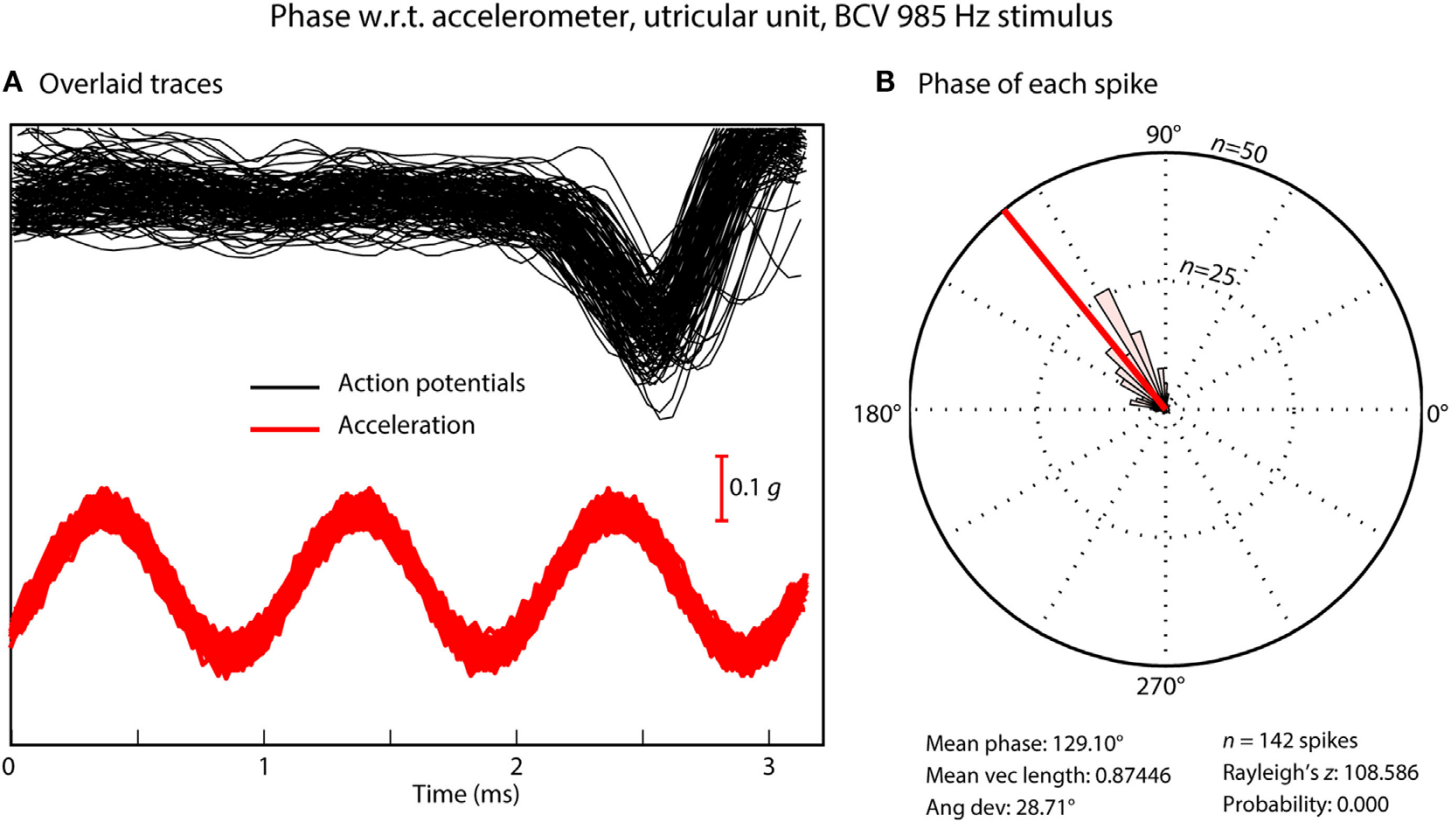
**(A)** Time series of action potentials in response to a bone-conducted vibration stimulus (shown by the red acceleration trace). Traces which contain a spike of neural firing are aligned using the timing of the stimulus pulse. **(B)** Circular histogram (rose plot) of the phase of each spike; the small and large concentric circles represent *n* = 25 and *n* = 50 spikes, respectively. The Rayleigh test of circular uniformity was performed on the 142 spikes, and was significant (*p* < 0.001), showing that the time when an afferent is activated is phase locked to the stimulus. Here, the neuron misses many cycles **(A)**, as can be seen from the value of the action potentials which contain no spikes in the cycles preceding each instance of firing, but the time when the neuron fires is locked to a narrow band of phase angles of the stimulus **(B)**. Clearly each individual cycle of the stimulus is acting to activate the receptor/afferent.

The exact response of these primary otolithic irregular neurons to BCV and ACS reveals a vital principle in the mechanism of transduction of high frequencies. For all neurons activated by ACS or BCV, the neurons do not fire an action potential on every single cycle, but the moment when the neuron fires is locked to a narrow band of phase angles of the stimulus waveform (Figure 4) (31, 38, 53). This is true up to very high frequencies even >3,000 Hz. For individual afferents, the measured optimum phase angle systematically changes with frequency for both ACS and BCV, reflecting the latency of the afferent. Also the optimum phase angle for an individual afferent neuron at a given frequency is not constant but varies from neuron to neuron (38, 53).

The phenomenon of phase locking shows that for both BCV and ACS, every single cycle of the sine wave stimulus is the effective stimulus for the afferent (31), even up to frequencies of 3,000 Hz where the duration of an individual cycle is so short (0.3 ms). It means that the receptors are being displaced at this very high frequency (3,000 times/s in this example), but when they fire is tightly locked to a particular phase angle of the sine wave stimulus even at this high frequency (38). Phase locking is very well established for cochlear receptors and afferents—the action potentials in cochlear afferent neurons are locked to each displacement of the basilar membrane. Phase locking of cochlear afferents is recognized as being a major code for the transmission of auditory frequency information (54, 55), see Fettiplace (56) for a recent excellent review. It is now clear that phase locking applies to otolithic neurons with very tight locking to particular phase angles up to high frequencies. This may be due to the fact that irregular afferents are excellent detectors of change in stimulation (jerk detectors) (31, 38).

**Figure 5:**
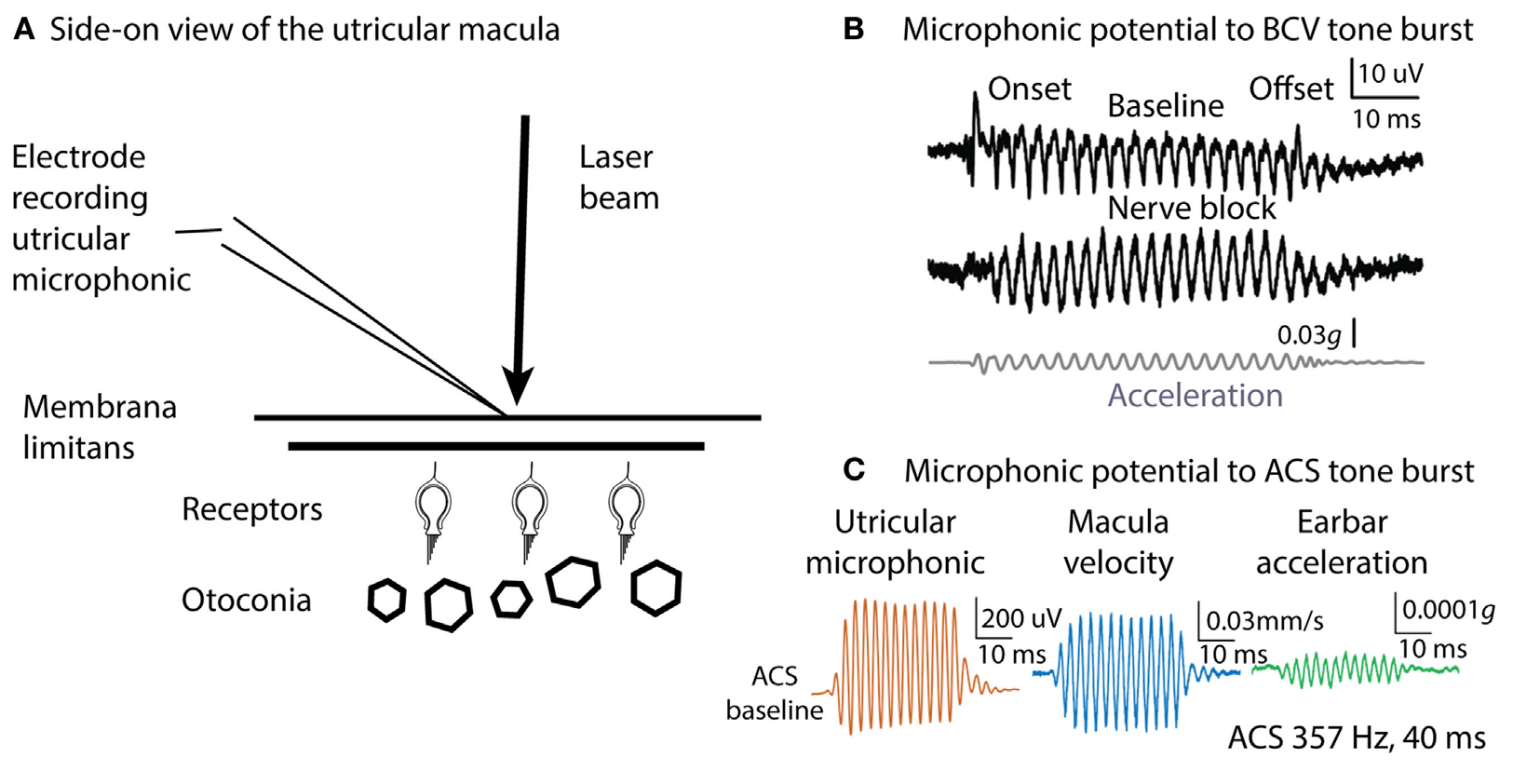
Microphonic recording and laser Doppler measurement of macula movement, showing the relation between the vestibular microphonic and the velocity of macula movement during bone-conducted vibration (BCV) or air-conducted sound (ACS) stimulation. A microelectrode on the surface of the utricular macula **(A)** records a microphonic potential from the utricular receptors in response to BCV **(B)** or to ACS **(C)**. There is no contribution from the cochlea since it has been completely ablated. **(B)** Vestibular microphonic responses to a BCV tone burst (40 ms, 400 Hz sinusoid) prior (top trace) and following (middle trace) lignocaine application to the vestibule to block the vestibular nerve. The microphonic remains after lignocaine injection showing it is a receptor field potential. Bottom trace: the BCV stimulus; the linear acceleration as recorded by a triaxial linear accelerometer on the ear bar. **(C)** Laser Doppler vibrometry. A laser beam is projected onto a reflective glass bead on the macula and the Doppler shift of the wavelength of the reflected beam shows the velocity of macula movement during BCV or ACS stimulation. Panel **(C)** shows the simultaneous measurement of vestibular microphonic and macula velocity. **(B)** Reprinted from Pastras et al. (57), © 2017, with permission from Elsevier. Panel **(C)** is from Pastras et al. (59).

How can such an apparently sluggish system as the otoliths with such dense otoconia exhibit phase locking to stimulus frequencies of thousands of Hertz? One answer comes from recording the vestibular microphonic, which shows that mammalian utricular receptors are activated at such high frequencies (57, 58). The vestibular microphonic is a field potential to sound or vibration and is a direct electrophysiological indicator of otolithic receptor hair cell function. The vestibular microphonic has been recorded *in vivo* in anesthetized guinea pigs by electrodes piercing the underside of the utricular macula with a glass microelectrode and then measuring the vestibular microphonic to BCV or ACS stimuli of varying frequency and amplitude (Figure 5) (57). Most importantly, in these animals, the cochlea has been completely removed, so there is no contribution from the cochlear microphonic. The recent paper (57) gives the evidence that the vestibular microphonic is a field potential due to otolithic receptor hair cell activation—reporting all the correct controls—such as chemically silencing afferent neurons and showing that the vestibular microphonic remains, and conversely chemically silencing the receptors and showing that the vestibular microphonic disappears, leading to the conclusion that the vestibular microphonic is a field potential generated by otolithic hair cells (utricular hair cells in this case) (Figure 5). The vestibular microphonic (strictly the utricular microphonic) has been recorded up to frequencies of 3,000 Hz. Those results complement the results from single neuron recordings: mammalian utricular receptors really do respond to very high frequencies (up to 3,000 Hz), far above what the otoliths are usually thought to be capable of transducing. But how? The simple answer is that the macula moves.

Many years ago, Tullio used fine aluminum particles on the surface of the utricular macula to demonstrate visually that sound caused rabbit utricular macula to move (60). We have confirmed Tullio’s results by using laser Doppler vibrometry to measure the velocity of guinea pig utricular macula movement during ACS and BCV stimulation. A tiny glass bead was placed on the exposed underside of the utricular macula and a laser beam aimed at it. The Doppler shift in the wavelength of the reflected beam during BCV or ACS stimulation (59) confirms that the macula is moving and gives the macula velocity. These measures show that both ACS and BCV cause the macula to move as Tullio had reported, and at frequencies up to 3,000 Hz. The actual displacements are small—nanometers—but the results of Geleoc et al. (52) show how very sensitive vestibular receptors are, so that deflections of the macula of nanometers can activate vestibular receptors.

Irregular otolithic afferents respond to the time rate of change of acceleration (jerk) rather than to acceleration itself. That jerk sensitivity has been demonstrated in otolithic evoked potentials (61) to pulses of linear acceleration. That jerk sensitivity adds to the puzzle—now this “sluggish” system not only transduces maintained linear accelerations but also this evidence shows it really does respond to extremely fast stimuli. The puzzle to be explained is that the one sensory system is responding over a large range of frequencies—from DC up to 3,000 Hz. How could high frequencies of BCV cause macula and hair bundle displacements at 3,000 Hz, given the very large specific gravity of the otoconia and the viscosity of the otolithic membrane?

**Figure 6:**
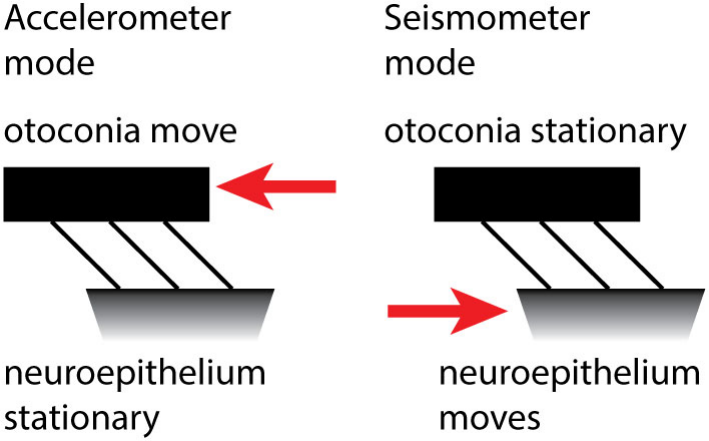
The accelerometer–seismometer model of otolith operation holds that at low frequencies *(left)* the otoconia move relative to the macula, but at high frequencies *(right)* the otoconia remain stationary while the macula moves. In both cases, the hair bundles are deflected and the receptors are activated.

Grant and Curthoys (62) have put forward a model of the otoliths which addresses that question. The model holds that there are two modes of otolithic operation: the traditional accelerometer mode and the new seismometer mode. At low frequencies of BCV, the otoconia move relative to the skull, while the macula stays stationary, and so the hair bundles of the receptor cells are deflected (Figure 6). This is the “traditional” accelerometer mode of operation. In the accelerometer mode, the otoconia move relative to the macula, while the macula is accelerated with the skull motion. At high frequencies, the system operates in the seismometer mode: the otoconia remain at rest (due to their inertia) while the macula is in motion, again producing a relative displacement between the otoconia and macula. In both cases, there is relative motion between the otoconia and macula, displacing hair bundles. We explain this in more detail below.

Otoliths are biological–mechanical sensors that measure the acceleration of the head in the plane of the otolith. The acceleration that is measured is the vector sum of gravity and the inertial acceleration and is called the gravitoinertial acceleration, but is generally just referred to as the head acceleration. The otolith acceleration value is a measurement of the relative displacement between the otolithic membrane and the NEL. This displacement measurement is made by the hair cells in the NEL and reported to the brain *via* the otolithic afferents.

Static or low-frequency linear acceleration causes the otoconia to move relative to the NEL of the utricular macula. During a maintained head tilt (a DC stimulus), the linear acceleration of gravity acts on the otoconia and displaces the otoconia relative to the NEL, so the hair bundles of the otolithic receptor hair cells (both type I and type II receptors) are deflected relative to their cell bodies and a neural signal is transmitted to the brain *via* the otolithic afferents, signaling that linear acceleration has occurred. This is the “traditional” accelerometer mode of otolith operation.

If a high-frequency vibration (e.g., 2,000 Hz) is applied to the skull, it causes the NEL to move at the same 2,000 Hz frequency. But because of their mass, the otoconia remain stationary. The consequence is that again the hair bundles will be deflected and action potentials will be propagated in otolithic afferent neurons. This is the seismometer mode of otolith operation. The difference is that in the first (accelerometer) mode, the otoconia move relative to the skull and in the second (seismometer) mode, the macula moves relative to the skull. In both modes, the otoconia and macula move with respect to each other, so the hair bundles of the receptors are displaced relative to the cell body. In this way, linear acceleration and high-frequency vibration can both stimulate the otolithic receptors.

Neurons cannot fire at such high rates (2,000 spikes/s), but at all frequencies the hair bundles of the receptors are deflected and activated once per cycle, and the neural evidence shows that when the afferent neurons fire, the action potentials show phase locking to the individual cycles of the stimulus at both low and high frequencies.

Given the usual stimulus strength used in VEMP testing to BCV, we estimate that the magnitude of these deflections is probably in the 50–80 nm range. With such small deflections, it is only the type I hair cells in the striolar region, that are stimulated. These type I hair cells are stiff (45) due to their large number of stereocilia and are stimulated with these small displacements seen in the high-frequency seismometer mode stimulus. The type II hair cells are less stiff and require larger deflections for stimulation. Afferents with regular resting discharge receive input predominantly from type II receptors, but are not activated by high-frequency BCV or ACS at the levels tested experimentally.

The model is essentially the result of application of engineering principles for the design of accelerometers and seismometers, to the otoliths. Importantly, engineering analysis shows that the one system can operate both as an accelerometer and as a seismometer. On this “accelerometer–seismometer” model, the one sensory system, the otoliths, transduces both low-frequency (even DC) linear accelerations and also very high-frequency stimuli. The empirical evidence that this happens comes from recordings of single otolithic afferents to a wide range of frequencies varying from 37 to 2,000 Hz (31) and showing that the one afferent is activated by stimuli across such a large frequency range, and from measuring (and modeling) the stimulus thresholds needed to activate the neuron across this large range.

Commercial accelerometers have an undamped natural frequency in the 10–20 kHz range and seismometers in the 5–10 Hz range. Otoliths have undamped natural frequencies in between these frequencies, which allows them to operate in both modes (accelerometer and seismometer) over the frequency ranges that have been shown to activate otoliths. It is the unique undamped natural frequency that allows the otoliths to make the transition over the two operating modes.

While this model accounts for the fact that receptor hair bundles can be displaced at various frequencies, we need to drill down into the micromechanics of hair bundle deflection to answer the final question: exactly how do the hair bundle deflections occur for both BCV and ACS stimuli? This comes down to what happens at the interface between the receptor cilia and the otolithic membrane during stimulation.

At the striola the short, stiff hair bundles of the receptors project into holes in the otolithic membrane (49, 50, 63–65). So any wall motion of the holes in the column filament-gel layer structure of the otolithic membrane will produce endolymph fluid motion within the hole. In the striolar region, the hair cell bundles are only weakly attached at the top of the kinocilium (27), or not attached at all and are free standing (49). This fluid motion within the hole produces a drag force on the bundle, causing it to deflect. The fluid environment is so viscously dominated (Reynold’s numbers—the ratio of inertial to viscous forces of 10^−3^–10^−2^) that bundles move instantaneously with any fluid movement. In other words, this coupling of fluid motion to hair bundle is so strong that the hair bundle displacement follows the fluid displacement almost exactly. The viscous dominated environment results in bundle displacement matching fluid displacement almost exactly, so fluid displacement is synonymous with hair bundle displacement. This account would also apply to receptor activation by ACS, since the vibrometry shows that the utricular macula moves during high-frequency ACS as well as during BCV. In sum, we suggest that the actual stimulus causing hair bundle deflection is the fluid displacement around the cilia of the type I receptors (see Box 1).

Box 1 **Transduction model of Grant and Curthoys (62).**

1. The otolithic system is underdamped. The transition from accelerometer mode to seismometer mode would not take place if the system were not underdamped.
2. The transition from accelerometer to seismometer takes place at the system undamped natural frequency (estimated to be around 600 Hz for humans).
3. In the accelerometer mode, head acceleration causes the otoconial layer (OL) to lag behind the neuroepithelial layer (NEL), producing a relative displacement between NEL and OL. This relative displacement deflects receptor hair bundles which activates the receptors.
4. In the seismometer mode at high frequencies, the OL remains at rest due to its inertia and the NEL is in motion, again producing relative displacement between the two layers and so again activating receptors.
5. Using vestibular-evoked myogenic potential (VEMP) test frequencies and acceleration magnitudes, we estimate the relative displacement between the two layers is around 50–80 nm. This displacement is small but sufficient to stimulate the short, stiff, loosely attached type I hair cell bundles in the striolar regions, while not large enough to activate extrastriolar type II hair bundles.
6. The model has implications for clinical testing: the ideal stimulus for otoliths and thus VEMPs is one with a very rapid rise time since the otolithic receptors are jerk detectors. That agrees with animal experimental (61) and clinical data (98) (see below) that short rise times are optimal for eliciting ocular vestibular-evoked myogenic potentials. Modeling of the neural data (62) indicates 750 Hz is probably the optimum frequency for testing VEMPs.

## Physiology Relevant for Clinical Testing

**Figure 7:**
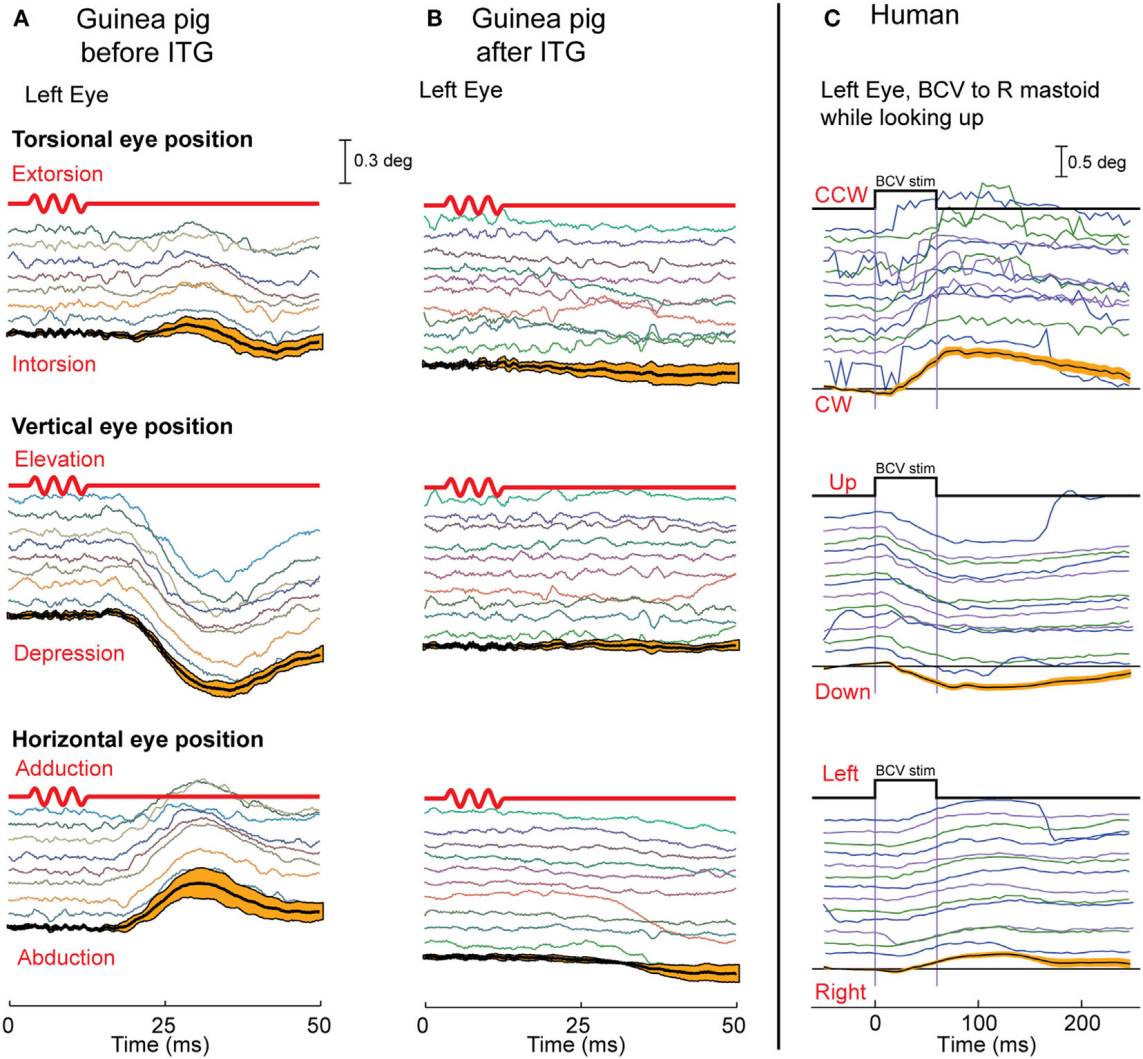
The eye movements in guinea pigs **(A,B)** and humans **(C)** in response to bone-conducted vibration (BCV). Each panel shows time series of torsional, vertical, and horizontal components of eye position in response to repeated tone bursts of 500 Hz BCV; below the traces are the mean and 95% confidence intervals *(orange bars)* calculated over responses to multiple stimuli. **(A,B)** The first line in red is the command voltage for the 500 Hz BCV stimulus. The eye movements in guinea pigs are eliminated **(B)** by intratympanic gentamicin which selectively attacks type I receptors. In humans **(C)**, a small vibration applied to the mastoid (start and end time shown by the top black trace) elicits stimulus-locked torsional, vertical, and horizontal eye movements. **(A,B)** Reprinted from Vulovic and Curthoys (67), © 2011, with permission from Elsevier.

Suzuki et al. electrically stimulated the utricular nerve in cats and showed it caused eye movements with torsional, vertical, and horizontal components (66). We reasoned that if 500 Hz BCV is a specific otolithic stimulus, it should generate a similar pattern of eye movements to those reported by Suzuki et al., and Vulovic and Curthoys (67) showed that brief 500 Hz BCV pulses of the skull of an alert guinea pig generated eye movements with horizontal vertical and torsional components similar to those Suzuki et al. found (Figure 7). These eye movements are due to vestibular as opposed to cochlear activation, because after intratympanic injection of gentamicin to the guinea pig, a procedure which selectively kills vestibular type I receptor cells (68, 69), the BCV evoked eye movements disappear but the indicator of cochlear function, the ABR response, remains (67).

Do these conclusions apply to human otolith-induced eye movements? In some healthy subjects (without any symptoms of superior canal dehiscence) we used fast high resolution video recording to record eye movements, and found that brief bursts of 500 Hz BCV of one mastoid delivered by a small clinical bone oscillator (Radioear B-71) caused small but systematic and reliable stimulus-locked eye movement responses with horizontal, vertical, and torsional components (Figure 7) at a short latency of about 20 ms or less (70). In these experiments, the subjects were biting on a bite-bar during the BCV stimulation to minimize head rotation and so minimize semicircular canal stimulation. Prior to such eye movements there would be electromyographic (EMG) potentials in the ocular muscles to cause the eye movement response, and it is these potentials in eye muscles which are recorded in VEMPs.

**Figure 8:**
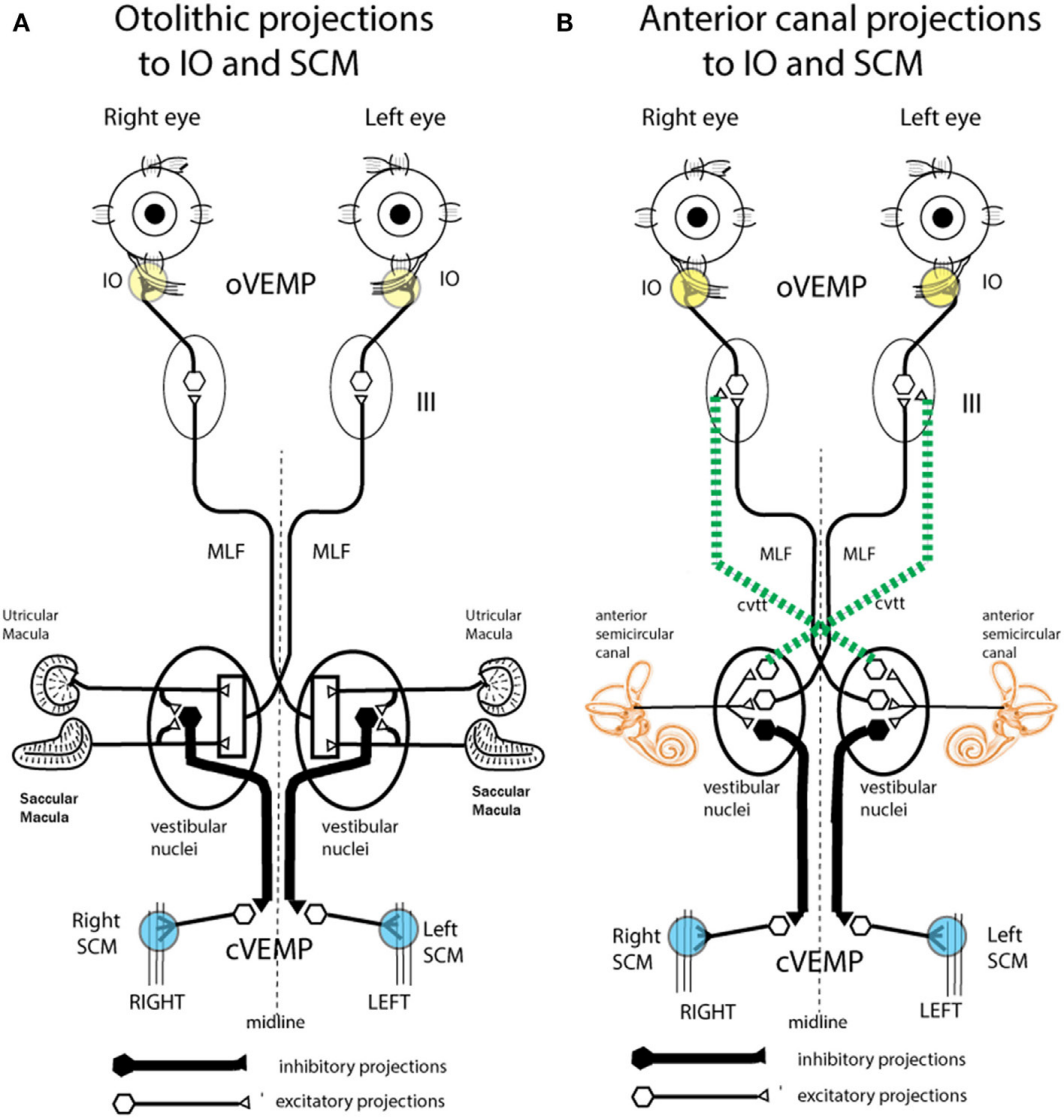
Schematic representations of the major neural projections from vestibular receptors to the eye muscles and the neck muscles. **(A)** The otolithic projections to inferior oblique eye muscle (IO) and sternocleidomastoid muscle (SCM). **(B)** The analogous projections of the anterior semicircular canal neurons to the IO and SCM (72). Stimulation in animals with intact labyrinths causes the neural connections shown on the left panel to be activated. However, after a semicircular canal dehiscence (SCD), the anterior semicircular canals are also activated by sound and vibration, so the neural projections on the right come into play. The green dotted lines represent the projection from the anterior canal neurons in the vestibular nucleus to the contralateral third nerve nucleus *via* the crossed ventral-tegmental track. It appears that it is this combination of otolithic and canal afferent activation which in part results in the enhanced ocular vestibular-evoked myogenic potential (oVEMP) and cervical vestibular-evoked myogenic potential (cVEMP) responses after SCD. **(A)** Reprinted by permission from John Wiley and Sons, Curthoys et al. (80), © 2011. **(B)** Reprinted by permission from Springer Nature, Curthoys (81), © 2017.

Air-conducted sound and BCV both activate both utricular and saccular afferents (29, 31, 33). Saccular afferents in guinea pigs do have a lower threshold to ACS than utricular afferents. But afferents from both maculae respond to both ACS and BCV (30, 31, 33). So how then is it possible to differentially assess utricular as opposed to saccular function? Curthoys put forward the original idea that the differential assessment of utricular and saccular function can come from the largely differential neural projections of these two systems (71). Physiology shows that short latency saccular projections to inferior oblique are weak, whereas saccular projections to neck and spinal motoneurons are strong (72). The work of Suzuki et al. (66) had shown that utricular projections to inferior oblique are strong, so Curthoys suggested that measuring the contralateral oVEMP—from the inferior oblique eye muscles—largely reflects the activation of contralateral utricular afferents by either ACS or BCV. Saccular projection to ipsilateral neck motoneurons is strong, so it was suggested that measuring the ipsilateral cVEMP from stretched neck muscles shows largely ipsilateral saccular function (71) (Figure 8). In this way, VEMPs can provide tests of utricular and saccular function not because of stimuli which selectively activate one or other of the two maculae, but by measuring responses which are predominantly determined by the differential neural projection of utricular as opposed to saccular projections to various muscle groups (Figure 8). This suggestion caused considerable controversy at the time (73, 74); however, data from patients with partial unilateral vestibular neuritis have provided evidence confirming those suggestions. In response to ACS or BCV some patients show *selective* loss of the contralateral oVEMP n10, but preservation of the ipsilateral cVEMP p13–n23 (75, 76). Other patients show the converse: symmetrical oVEMPs but asymmetrical cVEMPs: the ipsilateral cVEMP is reduced or eliminated, yet the oVEMP is not detectably affected (77). The logical consequence of that dissociation is that the two responses, oVEMP and cVEMP, must be generated from different sense organs—because to the same stimulus one response is affected, the other is not. Since the utricular afferents travel in the superior nerve and project to contralateral inferior oblique, it is most likely the utricular afferents which are affected. In light of these results, the Curthoys (71, 74) suggestion is now accepted: “Ocular vestibular evoked potentials are mainly dependent on utricular pathway function” [(78), p. 1843] and “The oVEMP originates predominantly from utricular afferents” [(79), p. 1051].

The stimulus frequency usually used for clinical testing of VEMPs is 500 Hz, and that frequency causes fairly selective activation of otolithic irregular neurons: at 500 Hz semicircular canal afferents with irregular resting discharge are not usually activated by sound or vibration in animals with normally encased bony labyrinths, at least up to BCV stimulus levels of 2 *g* or 130 dB SPL ACS (28, 31). Carey et al. reported that to elicit phase locking in irregular canal afferents in the chinchilla with a normally encased labyrinth required an extremely high intensity (135 dB SPL) (82). We have confirmed that result in guinea pigs (81, 83). Regular canal and otolith afferents are not activated usually by physiological levels of ACS and BCV. Higher level stimuli may cause them to be activated, but such levels are not clinically realistic. So usually there is little or no contribution from regular or irregular semicircular canal afferents during VEMP testing. That is changed in patients with a thinning—a dehiscence or window (fenestra or SCD) of the bony wall of the semicircular canal, who show very large VEMP potentials (discussed below).

The physiological results show that an SCD changes the neural response. After making an opening into the bony wall of the anterior canal, the procedure resulted in phase-locked activation of irregular canal afferents at a much lower intensity (96 dB SPL) than with the labyrinth encased (135 dB SPL) (82). These SCD-enhanced vestibular neural responses are consistent with the results from patients with a CT-verified SCD who show enhanced VEMPs to sound and vibration and nystagmus in the plane of the dehiscent canal during maintained tonal stimulation (84). However, it should be emphasized that both the neural and clinical results are variable; not all patients with CT-verified SCD develop the same classic symptoms, and there is considerable variability in the neural results (85). This is not surprising since the fenestra varies from patient to patient in humans and animals, and many other factors have the potential for influencing the results, such as collapse of the membranous duct (86).

**Figure 9:**
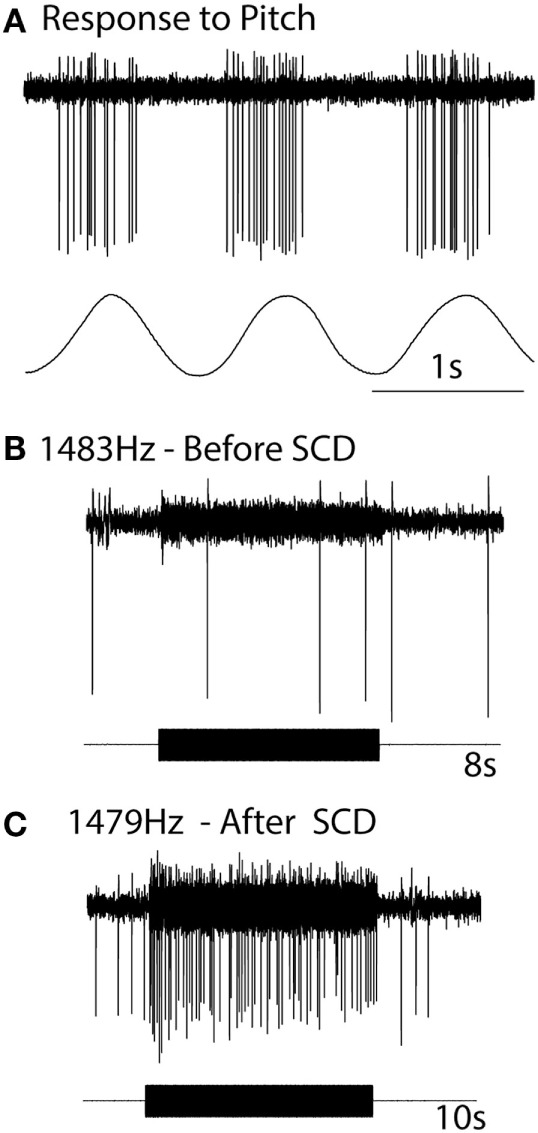
The response of the one anterior canal neuron to high-frequency air-conducted sound (ACS), before and after a small dehiscence in the bony wall of the anterior canal. **(A)** The response of the neuron to pitch angular acceleration identifies the neuron as being an anterior canal afferent. **(B)** Before semicircular canal dehiscence (SCD), an 8 s burst of 1,483 Hz ACS has no effect on the neural response. **(C)** After SCD, a 10 s burst of an ACS of 1,479 Hz causes strong activation. Resealing the SCD causes that enhanced response to disappear. Reprinted by permission from Springer Nature, Curthoys (81), © 2017.

The definitive evidence about the neural response in SCD comes from the response of individual neurons where the same neuron was recorded both before and after the SCD and in some cases after resealing the SCD (Figure 9). After SCD, guinea pig irregular semicircular canal afferents, previously unresponsive to ACS or BCV in animals with fully encased labyrinths, respond vigorously with low threshold to the same stimulus magnitude which was ineffective before SCD (83, 87). Maintained sound or vibration results in a maintained high firing rate in irregular anterior canal neurons. This has been confirmed by Iversen et al. (88), who also confirmed the report by Curthoys and Grant (53) that an SCD causes a slow change in firing of regular canal afferents to maintained sound. This change in neural firing corresponds to the cupula deflection caused by endolymph movement due to the SCD causing an impedance pumping type of action. Such a high firing rate would cause a maintained nystagmus in human patients (the Tullio phenomenon) (81). Nystagmus caused by such phase-locked activation in human patients would be expected to have abrupt onset and offset, as in fact happens in some patients in the clinical test called vibration-induced nystagmus (89).

Why should an SCD cause semicircular canal neurons previously unaffected by ACS or BCV now to respond to ACS and BCV? The SCD is a third window and so ACS and BCV cause larger fluid displacement in the duct (90, 91), and irregular canal afferents synapsing on type I receptors at the crest of the crista (43, 92, 93) are activated by these fluid displacements (83). Although similar structurally and physiologically to otolith type I receptors, canal type I receptors are not usually activated by ACS or BCV, because the sealed bony wall of the canal limits the amplitude of the sound-induced or vibration-induced fluid displacement in the canal. We reasoned that after an SCD, the increased fluid displacement is sufficient to deflect the short stiff cilia of type I semicircular canal receptors on the crista, and so irregular canal afferents would show phase-locked activation after SCD to both ACS and BCV, and in humans, canal neurons would thus contribute to the VEMP response as was found (83).

**Figure 10:**
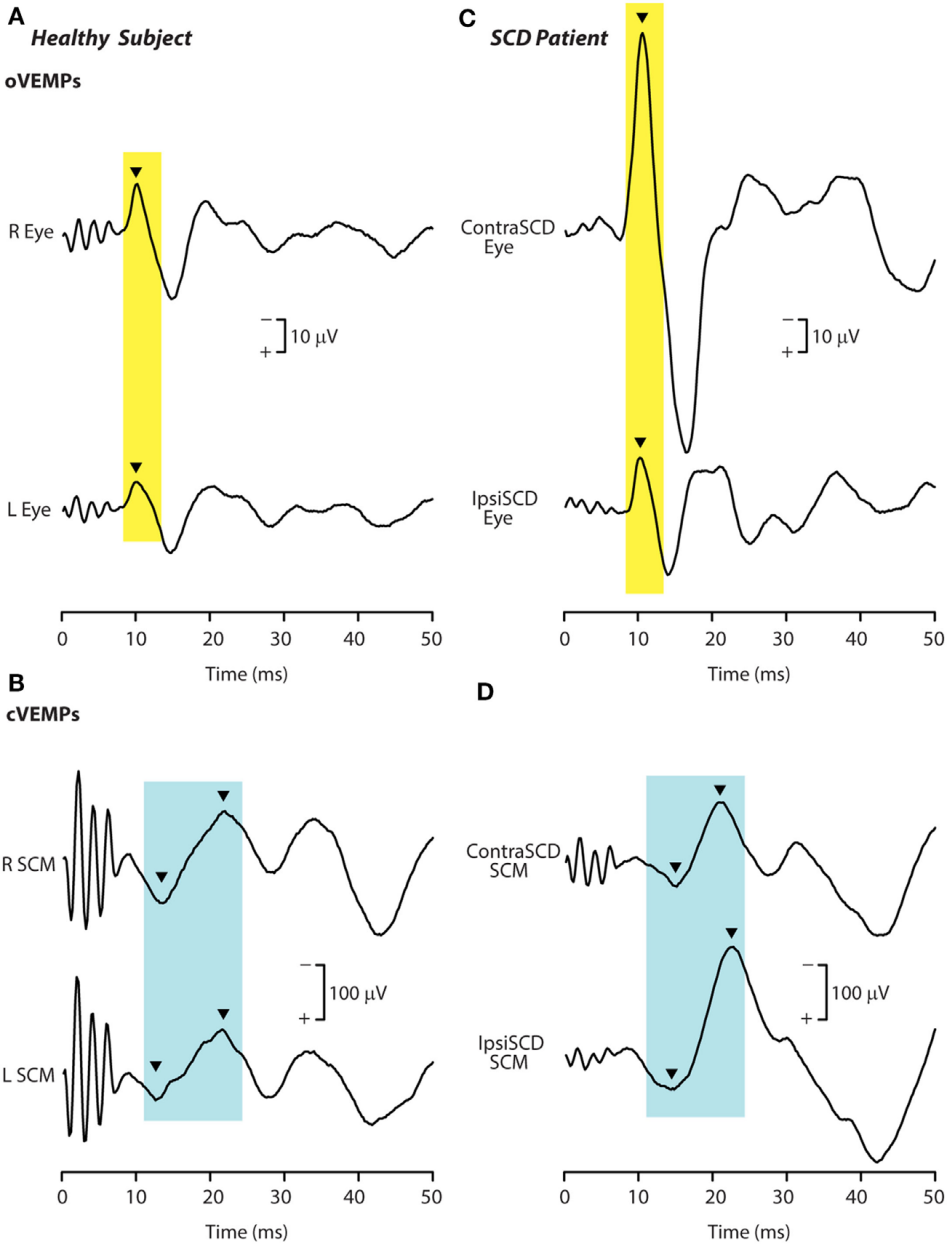
Recordings of ocular vestibular-evoked myogenic potentials (oVEMPs) **(A,C)** and cervical vestibular-evoked myogenic potentials (cVEMPs) **(B,D)** to 500 Hz bone-conducted vibration (BCV) from a healthy subject **(A,B)** and a patient with semicircular canal dehiscence (SCD) **(C,D)**. In each record, the stimulus onset occurred at time 0. In the healthy subject, BCV at the midline of forehead at the hairline (Fz) causes symmetric oVEMP beneath both eyes, with approximately equal amplitude oVEMP n10 components (arrowheads). By contrast, the same Fz stimulus causes an asymmetric n10 component of the oVEMP response in the patient: the oVEMP n10 recorded from beneath the contralesional eye is much larger than the oVEMP n10 recorded from beneath the ipsilesional eye, and is also much larger than seen in the healthy subject. In response to BCV at Fz, both subjects show clear cVEMP p13–n23 [arrowheads in **(B,D)**]. The response in the ipsilesional sternocleidomastoid muscle (SCM) in the patient is larger than in the patient’s contralesional SCM, but the asymmetry is not as great as in the same patient’s oVEMP traces. The cVEMP responses of the normal subject are more symmetrical than in the SCD patient. Reprinted by permission from Wolters Kluwer Health, Inc.: Manzari et al. (94), © 2012.

Such an outcome in human SCD patients would result in lower VEMP thresholds, as is observed. In addition, irregular anterior canal neurons project to contralateral inferior oblique, and so after SCD these neurons would now contribute to and enhance the oVEMP n10 response in the contralateral inferior oblique after SCD (Figure 10). Also by virtue of their ipsilateral (inhibitory) projection to SCM (72) (Figure 8), the activity of these canal afferents would enhance the cVEMP over the ipsilateral SCM.

In summary, the physiological evidence predicts the enhanced VEMP response seen after SCD. Patients with CT-verified SCD show VEMPs in response to very high-frequency stimulation which is ineffective in healthy subjects with intact bony labyrinth (95): a single VEMP test using 4,000 or 8,000 Hz elicits clear oVEMPs in such patients. Patients may have trouble even hearing the 8,000 Hz stimulus, which produces clear oVEMP n10 (95).

Afferents from other canals would probably not be affected by the SCD in one canal because after SCD the enhanced fluid displacement is apparently mainly directed to the canal with the new “third window.” This is in accord with what is usually found with human patients—the nystagmus produced by sound usually aligns with the canal in which the fenestra is located (96).

**Figure 11:**
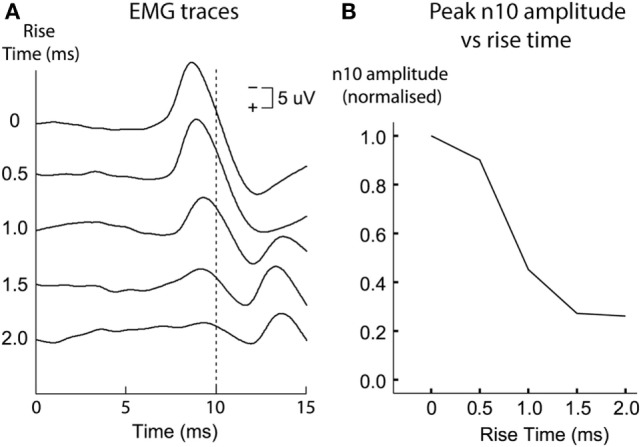
Ocular vestibular-evoked myogenic potential (oVEMP) responses [**(A)** time series for each stimulus type; **(B)** amplitudes of the n10 peak] from a subject receiving 500 Hz bone-conducted vibration stimuli at the midline of forehead at the hairline. Tone bursts of varying rise times (ramps) were presented in random order. The size of the n10 component of the oVEMP depends on the rise-time of the tone burst stimulus: increasing the rise time causes a systematic reduction of the n10 amplitude. This is quantified in panel **(B)**, where n10 amplitude is plotted against rise-time. Reprinted by permission from Wolters Kluwer Health, Inc.: Burgess et al. (98), © 2013.

So how is an oVEMP n10 in human subjects to ACS or BCV normally caused? Probably the most effective otolithic stimulus is a light tap with a tendon hammer to the skull at the midline of forehead at the hairline, because that is a high-jerk stimulus (97), and that pulse of jerk would be expected to cause simultaneous activation in many otolithic irregular afferents. We know it is the very onset of the stimulus which is effective in generating human oVEMP n10. Using very short rise-times (ramps) increases the magnitude of the oVEMP n10 (98) (Figure 11). Also, if a long duration 500 Hz stimulus is used and then its duration progressively reduced, the size of the oVEMP n10 for a stimulus duration of 2 ms is about the same as for a stimulus lasting 10 ms (99). The paradoxical aspect is that such a short stimulus sounds (and feels) pathetically weak, but the EMG measures show it is just as effective at eliciting an oVEMP n10 response as a long duration 10 ms stimulus (99) which subjectively appears to be a much stronger stimulus. Both results point to the very onset of the stimulus as being of great importance in determining the size of the oVEMP n10.

The widespread use of these tests together with the use of the video head impulse test of the function of all the semicircular canals has refined vestibular diagnosis of peripheral vestibular disorders. It is now clear that some patients have normal semicircular canal function bilaterally but total loss of otolith function unilaterally—as shown by reduced or absent oVEMP and cVEMP from one labyrinth. Even with unilateral loss of just the utricular macula with completely normal canal function and normal saccular function (100). These selective deficits support the contention that any individual sense organ of the vestibular labyrinth can exhibit dysfunction while the remainder of the labyrinth functions normally.

There is strong physiological evidence underpinning the initial step of VEMP—the activation of vestibular receptors by sound or vibration. The extensive projection of vestibular nuclei allow for many VEMP to be recorded. These with very short latency (cVEMPs and oVEMPs) have been favored for clinical evaluation of otolithic function (see Box 2 for summary).

Box 2 **Summary.**

- There are two kinds of otolithic receptor hair cells—amphora-shaped type I receptors and barrel-shaped type II receptors—and they are intermingled across the utricular and saccular maculae. There is a special band of receptors called the striola, on both the utricular macula and the saccular macula, where there is an increased concentration of receptors with short stiff cilia and poor attachment to the overlying otolithic membrane.
- Afferent neurons from the striolae form calyx synapses on type I receptors and have irregular resting discharge. It is these afferents which are activated by both sound and vibration. In animals with normally encased bony labyrinths, neurons with regular resting discharge are not activated by ACS or bone-conducted vibration (BCV) up to the maximum levels which were used.
- Bone-conducted vibration is a much more effective and reliable stimulus—vestibular neural thresholds to BCV are around the level for auditory brainstem response (ABR) threshold, whereas vestibular neural thresholds for ACS are around 70 dB above ABR threshold.
- The action potentials in the irregular afferents activated by sound and vibration are phase-locked to a particular band of phase angles of the stimulus waveform, up to frequencies well above 1,000 Hz for both ACS and BCV. In order to elicit this tight phase locking, each cycle of the waveform is the effective stimulus—each cycle is deflecting the hair bundles of the receptors.
- Grant and Curthoys (62) have suggested that the utricular macula operates both as an accelerometer at low frequencies and as a seismometer at high frequencies. On this model, at low frequencies the otoconia move relative to the receptor cell body (accelerometer mode), but at high frequencies the otoconia are stationary and the receptors move relative to the otoconia (seismometer mode).
- In both cases, the hair bundles are deflected relative to the cell body, so the receptors are activated both at low (accelerometer) and at high (seismometer) frequencies. That is confirmed by recording of the field potential of the utricular receptor hair cells—the utricular microphonic—which shows that utricular receptors are activated by the BCV stimulus up to high frequencies. It is stressed that the vestibular microphonic occurs without any input from the cochlea.
- Direct measures of utricular macula movement show that the macula moves up and down during vibration stimulation (and sound) up to frequencies of thousands of Hertz. The movements are very small, but *in vitro* studies (52) have shown that individual vestibular receptors have thresholds of nanometers of displacement, similar to the thresholds of cochlear receptors.
- In this way, 500 Hz mastoid vibration activates otolithic receptors and results in eye movements with horizontal, vertical, and torsional components, in human subjects consistent with utricular nerve activation at such high frequencies.
- Surface electrodes over muscle groups can record the electromyographic potentials evoked by abrupt simulation by ACS and BCV, and thus these vestibular evoked myogenic potentials (VEMPs) are being used in the clinic to indicate otolithic function.
- Because of the largely differential projection of the utricular macula to eye muscles and of the saccular macula to neck muscles, it has been possible to index predominantly utricular function by measuring the ocular vestibular-evoked myogenic potential by surface electrodes beneath the eyes as the subject looks up. Surface electrodes over tensed neck muscles record the cervical vestibular-evoked myogenic potential which indexes predominantly saccular function.

## Author Contributions

IC wrote the paper. JG wrote the section about the accelerometer–seismometer model. AB contributed to the section about clinical evidence. CP and DB contributed to the section about vestibular microphonics and vibrometry. LM contributed to the section about clinical evidence. All authors reviewed the text of the final paper.

## Conflict of Interest Statement

IC is an unpaid consultant to Otometrics, but has received support from Otometrics for travel and attendance at conferences and workshops. For the remaining authors the research was conducted in the absence of any commercial or financial relationships that could be construed as a potential conflict of interest.

## Abbreviations

ABR, auditory brainstem response; ACS, air-conducted sound; BCV, bone-conducted vibration; Fz, the midline of forehead at the hairline; IO, inferior oblique eye muscle; VEMP, vestibular-evoked myogenic potential; cVEMP, cervical vestibular-evoked myogenic potential; oVEMP, ocular vestibular-evoked myogenic potential; OL, otoconial layer; NEL, neuroepithelial layer; SCD, semicircular canal dehiscence; n10, the negative potential of the oVEMP at about 10 ms latency; SCM, sternocleidomastoid muscle; SPL, sound pressure level.

## References

[1] Weber KP Rosengren SM Clinical utility of ocular vestibular-evoked myogenic potentials (oVEMPs). Curr Neurol Neurosci Rep (2015) 15:22 10.1007/s11910-015-0548-y 25773001

[2] Dlugaiczyk J Ocular vestibular evoked myogenic potentials: where are we now? Otol Neurotol (2017) 38:E513–21. 10.1097/MAO.0000000000001478 29135871

[3] Clark B Graybiel A Perception of the postural vertical in normals and subjects with labyrinthine defects. J Exp Psychol (1963) 65:490–4. 10.1037/h0045606 14021495

[4] Diamond SG Markham CH Furuya N Binocular counter-rolling during sustained body tilt in normal humans and in a patient with unilateral vestibular nerve-section. Ann Otol Rhinol Laryngol (1982) 91:225–9. 10.1177/000348948209100222 6979286

[5] Graybiel A Oculogravic illusion. AMA Arch Ophthalmol (1952) 48:605–15. 10.1001/archopht.1952.00920010616007 12984891

[6] Lichtenberg BK Young LR Arrott AP Human ocular counter-rolling induced by varying linear accelerations. Exp Brain Res (1982) 48:127–36. 10.1007/BF00239580 7140883

[7] Miller EF Fregly AR Graybiel A Visual horizontal-perception in relation to otolith-function. Am J Psychol (1968) 81:488–96. 10.2307/1421053 5760030

[8] Schone H On the role of gravity in human spatial orientation. Aerosp Med (1964) 35:764–72. 14215796

[9] Rudisill HE Hain TC. Lower extremity myogenic potentials evoked by acoustic stimuli in healthy adults. Otol Neurotol (2008) 29:688–92. 10.1097/MAO.0b013e3181730377 18665033

[10] Cherchi M Bellinaso NP Card K Covington A Krumpe A Pfeifer MS Sound evoked triceps myogenic potentials. Otol Neurotol (2009) 30:545–50. 10.1097/MAO.0b013e31819d89eb 19373121

[11] Colebatch JG Halmagyi GM Skuse NF. Myogenic potentials generated by a click-evoked vestibulocollic reflex. J Neurol Neurosurg Psychiatry (1994) 57:190–7. 10.1136/jnnp.57.2.190 8126503 PMC1072448

[12] Rosengren SM Todd NPM Colebatch JG Vestibular-evoked extraocular potentials produced by stimulation with bone-conducted sound. Clin Neurophysiol (2005) 116:1938–48. 10.1016/j.clinph.2005.03.019 15979939

[13] Iwasaki S McGarvie LA Halmagyi GM Burgess AM Kim J Colebatch JG Head taps evoke a crossed vestibulo-ocular reflex. Neurology (2007) 68:1227–9. 10.1212/01.wnl.0000259064.80564.21 17420408

[14] Todd NP Rosengren SM Aw ST Colebatch JG Ocular vestibular evoked myogenic potentials (OVEMPs) produced by air- and bone-conducted sound. Clin Neurophysiol (2007) 118:381–90. 10.1016/j.clinph.2006.09.025 17141563

[15] Halmagyi GM Yavor RA Colebatch JG. Tapping the head activates the vestibular system: a new use for the clinical reflex hammer. Neurology (1995) 45:1927–9. 10.1212/WNL.45.10.1927 7477996

[16] Iwasaki S Chihara Y Smulders YE Burgess AM Halmagyi GM Curthoys IS The role of the superior vestibular nerve in generating ocular vestibular-evoked myogenic potentials to bone conducted vibration at Fz. Clin Neurophysiol (2009) 120:588–93. 10.1016/j.clinph.2008.12.036 19211301

[17] Colebatch JG Halmagyi GM Vestibular evoked potentials in human neck muscles before and after unilateral vestibular deafferentation. Neurology (1992) 42:1635–6. 10.1212/WNL.42.8.1635 1641165

[18] Maqsood A Anis-ur-Rehman M Gul IH Chemical composition, density, specific gravity, apparent porosity, and thermal transport properties of volcanic rocks in the temperature range 253 to 333 K. J Chem Eng Data (2003) 48:1310–4. 10.1021/je034077p

[19] Lopez I Ishiyama G Tang Y Frank M Baloh RW Ishiyama A. Estimation of the number of nerve fibers in the human vestibular endorgans using unbiased stereology and immunohistochemistry. J Neurosci Methods (2005) 145:37–46. 10.1016/j.jneumeth.2004.11.024 15922024

[20] Bergström B Morphology of the vestibular nerve. III. Analysis of the calibers of the myelinated vestibular nerve fibers in man at various ages. Acta Otolaryngol (1973) 76:331–8. 10.3109/00016487309121518 4543917

[21] Baloh RW Honrubia V Clinical Neurophysiology of the Vestibular System. 2nd ed Philadelphia, PA: F. A. Davis (1990). 378525

[22] DeVries H The mechanics of the labyrinth otoliths. Acta Otolaryngol (1950) 38:262–73. 10.3109/00016485009118384 14856657

[23] Grant W Best W Otolith-organ mechanics – lumped parameter model and dynamic-response. Aviat Space Environ Med (1987) 58:970–6. 3314853

[24] Cotton JR Grant JW A finite element method for mechanical response of hair cell ciliary bundles. J Biomech Eng (2000) 122:44–50. 10.1115/1.429626 10790829

[25] Dunlap MD Grant JW. Experimental measurement of utricle system dynamic response to inertial stimulus. J Assoc Res Otolaryngol (2014) 15:511–28. 10.1007/s10162-014-0456-x 24845403 PMC4141440

[26] Grant JW Best WA Lonigro R Governing equations of motion for the otolith organs and their response to a step change in velocity of the skull. J Biomech Eng (1984) 106:302–8. 10.1115/1.3138498 6513524

[27] Lindeman HH Studies on the morphology of the sensory regions of the vestibular apparatus with 45 figures. Ergeb Anat Entwicklungsgesch (1969) 42:1–113. 5310109

[28] Curthoys IS Kim J McPhedran SK Camp AJ. Bone conducted vibration selectively activates irregular primary otolithic vestibular neurons in the guinea pig. Exp Brain Res (2006) 175:256–67. 10.1007/s00221-006-0544-1 16761136

[29] Curthoys IS Vulovic V. Vestibular primary afferent responses to sound and vibration in the guinea pig. Exp Brain Res (2011) 210:347–52. 10.1007/s00221-010-2499-5 21113779

[30] Curthoys IS Vulovic V Burgess AM Manzari L Sokolic L Pogson J Neural basis of new clinical vestibular tests: otolithic neural responses to sound and vibration. Clin Exp Pharmacol Physiol (2014) 41:371–80. 10.1111/1440-1681.12222 24754528

[31] Curthoys IS Vulovic V Burgess AM Sokolic L Goonetilleke SC The response of guinea pig primary utricular and saccular irregular neurons to bone-conducted vibration (BCV) and air-conducted sound (ACS). Hear Res (2016) 331:131–43. 10.1016/j.heares.2015.10.019 26626360

[32] Curthoys IS Vulovic V Pogson J Sokolic L Responses of guinea pig primary vestibular afferents to low frequency (50-100 Hz) bone conducted vibration (BCV) – the neural basis of vibration induced nystagmus. Program No 57406 2012 Neuroscience Meeting Planner [Online]. New Orleans, LA: Society for Neuroscience (2013).

[33] Curthoys IS Vulovic V Sokolic L Pogson J Burgess AM. Irregular primary otolith afferents from the guinea pig utricular and saccular maculae respond to both bone conducted vibration and to air conducted sound. Brain Res Bull (2012) 89:16–21. 10.1016/j.brainresbull.2012.07.007 22814095

[34] Fernandez C Goldberg JM. Physiology of peripheral neurons innervating otolith organs of the squirrel monkey. I. Response to static tilts and to long-duration centrifugal force. J Neurophysiol (1976) 39:970–84. 10.1152/jn.1976.39.5.970 824412

[35] McCue MP Guinan JJ Jr. Acoustically responsive fibers in the vestibular nerve of the cat. J Neurosci (1994) 14:6058–70. 10.1523/JNEUROSCI.14-10-06058.1994 7931562 PMC6576982

[36] McCue MP Guinan JJ Jr Sound-evoked activity in primary afferent neurons of a mammalian vestibular system. Am J Otol (1997) 18:355–60. 9149831

[37] Murofushi T Curthoys IS Topple AN Colebatch JG Halmagyi GM. Responses of guinea pig primary vestibular neurons to clicks. Exp Brain Res (1995) 103:174–8. 10.1007/BF00241975 7615033

[38] Young ED Fernandez C Goldberg JM. Responses of squirrel monkey vestibular neurons to audio-frequency sound and head vibration. Acta Otolaryngol (1977) 84:352–60. 10.3109/00016487709123977 303426

[39] Zhu H Tang X Wei W Maklad A Mustain W Rabbitt R Input-output functions of vestibular afferent responses to air-conducted clicks in rats. J Assoc Res Otolaryngol (2014) 15:73–86. 10.1007/s10162-013-0428-6 24297262 PMC3901862

[40] Zhu H Tang X Wei W Mustain W Xu Y Zhou W. Click-evoked responses in vestibular afferents in rats. J Neurophysiol (2011) 106:754–63. 10.1152/jn.00003.2011 21613592

[41] Curthoys IS MacDougall HG Vidal PP de Waele C. Sustained and transient vestibular systems: a physiological basis for interpreting vestibular function. Front Neurol (2017) 8:117. 10.3389/fneur.2017.00117 28424655 PMC5371610

[42] Uzun-Coruhlu H Curthoys IS Jones AS. Attachment of the utricular and saccular maculae to the temporal bone. Hear Res (2007) 233:77–85. 10.1016/j.heares.2007.07.008 17919861

[43] Desai SS Zeh C Lysakowski A. Comparative morphology of rodent vestibular periphery. I. Saccular and utricular maculae. J Neurophysiol (2005) 93:251–66. 10.1152/jn.00746.2003 15240767 PMC12456082

[44] Watanuki K Schuknecht HF A morphological study of human vestibular sensory epithelia. Arch Otolaryngol (1976) 102:583–8. 10.1001/archotol.1976.00780150051001 1086086

[45] Spoon C Grant W. Biomechanics of hair cell kinocilia: experimental measurement of kinocilium shaft stiffness and base rotational stiffness with Euler-Bernoulli and Timoshenko beam analysis. J Exp Biol (2011) 214:862–70. 10.1242/jeb.051151 21307074 PMC3036549

[46] Watanuki K Meyer zum Gottesberge A Morphological observations of the sensory epithelium of the macula sacculi and utriculi in the guinea pig. Arch Klin Exp Ohren Nasen Kehlkopfheilkd (1971) 200:136–44. 10.1007/BF00418197 4107253

[47] Fernandez C Goldberg JM Baird RA. The vestibular nerve of the chinchilla. III. Peripheral innervation patterns in the utricular macula. J Neurophysiol (1990) 63:767–80. 10.1152/jn.1990.63.4.767 2341875

[48] Goldberg JM Desmadryl G Baird RA Fernandez C. The vestibular nerve of the chinchilla. IV. Discharge properties of utricular afferents. J Neurophysiol (1990) 63:781–90. 10.1152/jn.1990.63.4.781 2341876

[49] Lim DJ Morphological and physiological correlates in cochlear and vestibular sensory epithelia. Scan Electron Microsc (1976) 2:269–76.

[50] Lim DJ Fine morphology of the otoconial membrane and its relationship to the sensory epithelium. Scan Electron Microsc (1979) 3:929–38. 524064

[51] Songer JE Eatock RA. Tuning and timing in mammalian type I hair cells and calyceal synapses. J Neurosci (2013) 33:3706–24. 10.1523/JNEUROSCI.4067-12.2013 23426697 PMC3857958

[52] Geleoc GSG Lennan GWT Richardson GP Kros CJ. A quantitative comparison of mechanoelectrical transduction in vestibular and auditory hair cells of neonatal mice. Proc Biol Sci (1997) 264:611–21. 10.1098/rspb.1997.0087 9149428 PMC1688386

[53] Curthoys IS Grant JW In what way is an air conducted sound an otolithic stimulus? PS-927. Abstr Assoc Res Otolaryngol (2016) 39:568.

[54] Palmer AR Russell IJ. Phase-locking in the cochlear nerve of the guinea-pig and its relation to the receptor potential of inner hair-cells. Hear Res (1986) 24:1–15. 10.1016/0378-5955(86)90002-X 3759671

[55] Rose JE Brugge JF Anderson DJ Hind JE Phase-locked response to low-frequency tones in single auditory nerve fibers of the squirrel monkey. J Neurophysiol (1967) 30:769–93. 10.1152/jn.1967.30.4.769 4962851

[56] Fettiplace R. Hair cell transduction, tuning, and synaptic transmission in the mammalian cochlea. Compr Physiol (2017) 7:1197–227. 10.1002/cphy.c160049 28915323 PMC5658794

[57] Pastras CJ Curthoys IS Brown DJ In vivo recording of the vestibular microphonic in mammals. Hear Res (2017) 354:38–47. 10.1016/j.heares.2017.07.015 28850921

[58] Brown DJ Pastras CJ Curthoys IS. Electrophysiological measurements of peripheral vestibular function – a review of electrovestibulography. Front Syst Neurosci (2017) 11:34. 10.3389/fnsys.2017.00034 28620284 PMC5450778

[59] Pastras C Curthoys IS Brown D Dynamic response and sensitivity of the utricular macula, measured in vivo using laser Doppler vibrometry in guinea pigs. PS547. Abstr Assoc Res Otolaryngol (2018) 41:342. 10.1016/j.heares.2018.08.005 30170855

[60] Tullio P L’orecchio. Bologna: L. Capelli Editore (1928).

[61] Jones TA Jones SM Vijayakumar S Brugeaud A Bothwell M Chabbert C. The adequate stimulus for mammalian linear vestibular evoked potentials (VsEPs). Hear Res (2011) 280:133–40. 10.1016/j.heares.2011.05.005 21664446 PMC3826178

[62] Grant W Curthoys I. Otoliths – accelerometer and seismometer; implications in vestibular evoked myogenic potential (VEMP). Hear Res (2017) 353:26–35. 10.1016/j.heares.2017.07.012 28777976

[63] Lim DJ Vestibular sensory organs – scanning electron microscopic investigation. Arch Otolaryngol (1971) 94:69–76. 10.1001/archotol.1971.00770070105013 5555875

[64] Lim DJ Anniko M. Developmental morphology of the mouse inner ear. A scanning electron microscopic observation. Acta Otolaryngol Suppl (1985) 422:1–69. 10.3109/00016488509121766 3877398

[65] Lim DJ Lane WC Vestibular sensory epithelia – a scanning electron microscopic observation. Arch Otolaryngol (1969) 90:283–92. 10.1001/archotol.1969.00770030285007 5306613

[66] Suzuki JI Tokumasu K Goto K Eye movements from single utricular nerve stimulation in the cat. Acta Otolaryngol (1969) 68:350–62. 10.3109/00016486909121573 5309166

[67] Vulovic V Curthoys IS. Bone conducted vibration activates the vestibulo-ocular reflex in the guinea pig. Brain Res Bull (2011) 86:74–81. 10.1016/j.brainresbull.2011.06.013 21745548

[68] Lyford-Pike S Vogelheim C Chu E Della Santina CC Carey JP. Gentamicin is primarily localized in vestibular type I hair cells after intratympanic administration. J Assoc Res Otolaryngol (2007) 8:497–508. 10.1007/s10162-007-0093-8 17899270 PMC2538341

[69] Lue J-H Day A-S Cheng P-W Young Y-H. Vestibular evoked myogenic potentials are heavily dependent on type I hair cell activity of the saccular macula in guinea pigs. Audiol Neurootol (2009) 14:59–66. 10.1159/000156701 18812694

[70] Cornell ED Burgess AM MacDougall HG Curthoys IS. Bone conducted vibration to the mastoid produces horizontal, vertical and torsional eye movements. J Vestib Res (2015) 25:91–6. 10.3233/VES-150550 26410673

[71] Curthoys IS. A critical review of the neurophysiological evidence underlying clinical vestibular testing using sound, vibration and galvanic stimuli. Clin Neurophysiol (2010) 121:132–44. 10.1016/j.clinph.2009.09.027 19897412

[72] Uchino Y Kushiro K. Differences between otolith- and semicircular canal-activated neural circuitry in the vestibular system. Neurosci Res (2011) 71:315–27. 10.1016/j.neures.2011.09.001 21968226

[73] Colebatch JG Sound conclusions? Clin Neurophysiol (2010) 121:124–6. 10.1016/j.clinph.2009.09.026 19897411

[74] Curthoys IS A balanced view of the evidence leads to sound conclusions. A reply to J.G. Colebatch “Sound conclusions?” Clin Neurophysiol (2010) 121:977–8. 10.1016/j.clinph.2010.01.025 20181516

[75] Manzari L Tedesco A Burgess AM Curthoys IS Ocular vestibular-evoked myogenic potentials to bone-conducted vibration in superior vestibular neuritis show utricular function. Otolaryngol Head Neck Surg (2010) 143:274–80. 10.1016/j.otohns.2010.03.020 20647134

[76] Curthoys IS Iwasaki S Chihara Y Ushio M McGarvie LA Burgess AM The ocular vestibular-evoked myogenic potential to air-conducted sound; probable superior vestibular nerve origin. Clin Neurophysiol (2011) 122:611–6. 10.1016/j.clinph.2010.07.018 20709596

[77] Manzari L Burgess AM Curthoys IS Ocular and cervical vestibular evoked myogenic potentials in response to bone-conducted vibration in patients with probable inferior vestibular neuritis. J Laryngol Otol (2012) 126:683–91. 10.1017/S0022215112000692 22583739

[78] Papathanasiou ES The evidence is finally here: ocular vestibular evoked myogenic potentials are mainly dependent on utricular pathway function. Clin Neurophysiol (2015) 126:1843–4. 10.1016/j.clinph.2015.01.007 25703939

[79] Govender S Colebatch JG. Location and phase effects for ocular and cervical vestibular-evoked myogenic potentials evoked by bone-conducted stimuli at midline skull sites. J Neurophysiol (2018) 119:1045–56. 10.1152/jn.00695.2017 29357475

[80] Curthoys IS Vulovic V Burgess AM Cornell ED Mezey LE MacDougall HG The basis for using bone-conducted vibration or air-conducted sound to test otolithic function. Ann N Y Acad Sci (2011) 1233:231–41. 10.1111/j.1749-6632.2011.06147.x 21950999

[81] Curthoys IS. The new vestibular stimuli: sound and vibration-anatomical, physiological and clinical evidence. Exp Brain Res (2017) 235:957–72. 10.1007/s00221-017-4874-y 28130556

[82] Carey JP Hirvonen TP Hullar TE Minor LB. Acoustic responses of vestibular afferents in a model of superior canal dehiscence. Otol Neurotol (2004) 25:345–52. 10.1097/00129492-200405000-00024 15129116

[83] Curthoys IS Grant JW. How does high-frequency sound or vibration activate vestibular receptors? Exp Brain Res (2015) 233:691–9. 10.1007/s00221-014-4192-6 25567092

[84] Cremer PD Minor LB Carey JP Della Santina CC. Eye movements in patients with superior canal dehiscence syndrome align with the abnormal canal. Neurology (2000) 55:1833–41. 10.1212/WNL.55.12.1833 11134382

[85] Ward BK Carey JP Minor LB. Superior canal dehiscence syndrome: lessons from the first 20 years. Front Neurol (2017) 8:177. 10.3389/fneur.2017.00177 28503164 PMC5408023

[86] Manzari L Burgess AM MacDougall HG Curthoys IS. Enhanced otolithic function in semicircular canal dehiscence. Acta Otolaryngol (2011) 131:107–12. 10.3109/00016489.2010.507780 20863151

[87] Dlugaiczyk J Burgess AM Goonetilleke S Sokolic L Curthoys IS Superior canal dehiscence syndrome: relating clinical findings with vestibular neural responses from a guinea pig model. Otol Neurotol (2018) (in press). 10.1097/MAO.0000000000001940 30870375

[88] Iversen M Zhu H Zhou W Della Santina CC Carey J Rabbitt RD The biophysical origins of Tullio phenomenon. PS546. Abstr Assoc Res Otolaryngol (2018) 41:341–2.

[89] Dumas G Curthoys IS Lion A Perrin P Schmerber S. The skull vibration-induced nystagmus test of vestibular function – a review. Front Neurol (2017) 8:41. 10.3389/fneur.2017.00041 28337171 PMC5343042

[90] Chien W Rosowski JJ Ravicz ME Rauch SD Smullen J Merchant SN. Measurements of stapes velocity in live human ears. Hear Res (2009) 249:54–61. 10.1016/j.heares.2008.11.011 19111599 PMC2874870

[91] Rosowski JJ Songer JE Nakajima HH Brinsko KM Merchant SN. Clinical, experimental, and theoretical investigations of the effect of superior semicircular canal dehiscence on hearing mechanisms. Otol Neurotol (2004) 25:323–32. 10.1097/00129492-200405000-00021 15129113

[92] Desai SS Ali H Lysakowski A. Comparative morphology of rodent vestibular periphery. II. Cristae ampullares. J Neurophysiol (2005) 93:267–80. 10.1152/jn.00747.2003 15240768 PMC12513555

[93] Lysakowski A Goldberg JM. A regional ultrastructural analysis of the cellular and synaptic architecture in the chinchilla cristae ampullares. J Comp Neurol (1997) 389:419–43. 10.1002/(SICI)1096-9861(19971222)389:3<419::AID-CNE5>3.0.CO;2-3 9414004 PMC11468975

[94] Manzari L Burgess AM McGarvie LA Curthoys IS. Ocular and cervical vestibular evoked myogenic potentials to 500 Hz Fz bone-conducted vibration in superior semicircular canal dehiscence. Ear Hear (2012) 33:508–20. 10.1097/AUD.0b013e3182498c09 22441357

[95] Manzari L Burgess AM McGarvie LA Curthoys IS. An indicator of probable semicircular canal dehiscence: ocular vestibular evoked myogenic potentials to high frequencies. Otolaryngol Head Neck Surg (2013) 149:142–5. 10.1177/0194599813489494 23674567

[96] Cremer PD Migliaccio AA Pohl DV Curthoys IS Davies L Yavor RA Posterior semicircular canal nystagmus is conjugate and its axis is parallel to that of the canal. Neurology (2000) 54:2016–20. 10.1212/WNL.54.10.2016 10822450

[97] Iwasaki S Smulders YE Burgess AM McGarvie LA Macdougall HG Halmagyi GM Ocular vestibular evoked myogenic potentials to bone conducted vibration of the midline forehead at Fz in healthy subjects. Clin Neurophysiol (2008) 119:2135–47. 10.1016/j.clinph.2008.05.028 18639490

[98] Burgess AM Mezey LE Manzari L MacDougall HG McGarvie LA Curthoys IS. Effect of stimulus rise-time on the ocular vestibular-evoked myogenic potential to bone-conducted vibration. Ear Hear (2013) 34:799–805. 10.1097/AUD.0b013e318294e3d2 23732683

[99] Lim LJZ Dennis DL Govender S Colebatch JG. Differential effects of duration for ocular and cervical vestibular evoked myogenic potentials evoked by air- and bone-conducted stimuli. Exp Brain Res (2013) 224:437–45. 10.1007/s00221-012-3323-1 23161155

[100] Pelosi S Schuster D Jacobson GP Carlson ML Haynes DS Bennett ML Clinical characteristics associated with isolated unilateral utricular dysfunction. Am J Otolaryngol (2013) 34:490–5. 10.1016/j.amjoto.2013.04.008 23759133

